# The Magnetic Receptor of *Monascus ruber* M7: Gene Clone and Its Heterologous Expression in *Escherichia coli*

**DOI:** 10.3389/fmicb.2020.01112

**Published:** 2020-06-19

**Authors:** Hongyi Zhou, Shuyan Yang, Fusheng Chen

**Affiliations:** ^1^Hubei International Scientific and Technological Cooperation Base of Traditional Fermented Foods, Huazhong Agricultural University, Wuhan, China; ^2^College of Food Science and Technology, Huazhong Agricultural University, Wuhan, China

**Keywords:** *Monascus ruber*, magnetic receptor, blue light, heterologous expression, Western blot

## Abstract

It is well known that many organisms can perceive the magnetic field (MF), including the geomagnetic field, but how to feel MF is unclear. Recently, a study has claimed that a biological compass, namely a complex of the magnetic receptor (MagR) and blue light (BL) receptor (cryptochrome), has been found in *Homo sapiens*, *Drosophila melanogaster*, and *Danaus plexippus*, which may bring some new ideas to explore the mechanism of biomagnetism. *Monascus* spp. are edible filamentous fungi that can produce abundant beneficial secondary metabolites and have been used to produce food colorants for nearly 2000 years in the world, especially in China, Japan, and Korea. In this work, we firstly treated *M. ruber* M7 by BL (500 lux,465–467 nm), MF (5, 10, 30 mT), and the combination of MF and BL (MF-BL), respectively. The results revealed that, compared with the control (CK, neither BL nor MF), the MF alone had no effect on the growth and morphological characteristics of M7, but BL made the colonial diameters only 66.7% of CK’s and inhibited the formation of cleistothecia. Under MF-BL, the colony diameters were still 66.7% of CK’s, but the colonial growth and cleistothecia production inhibited by BL were partially restored. Then, we have found that the *magR* gene widely exists in the genomes of animals, plants, and microorganisms, and we have also discovered a *magR* gene in the M7 genome, hereinafter referred to *mr-magR*. Finally, the full-length cDNA of *mr-magR* was successfully cloned and expressed in *Escherichia coli* BL21 (DE3), and the Mr-MagR protein was purified by a Ni^+^-NTA column and identified by Western blot. These results have laid a foundation for further investigation on the relationship between Mr-MagR and BL receptor(s) that might exist in M7. According to a literature search, it is the first time to report *magR* in filamentous fungi.

## Introduction

Many organisms, including animals (Cain et al., 2005; Zhan et al., 2011; O’Neill, 2013), plants (Garcia and Arza, 2001; Zhadin, 2001; Anand et al., 2012), and microorganisms (Blakemore, 1975; Frankel et al., 1979; Faivre and Schuler, 2008; Rakoczy et al., 2016) can perceive the magnetic field (MF), including the geomagnetic field. To explain the phenomenon of biological MF perception, several hypotheses and models have been proposed (Frankel and Blakemore, 1980; Liboff et al., 1987; Ritz et al., 2000; Johnsen and Lohmann, 2005; Wiltschko, 2012). Among them, the magnetite-based model and the radical-pair-reaction-based chemical model (RCM) are probably the most well-researched hypotheses so far (Ritz et al., 2004; Mouritsen and Ritz, 2005; Maeda et al., 2008; Rodgers and Hore, 2009; Mouritsen and Hore, 2012; Giachello et al., 2016; Hore and Mouritsen, 2016; Nordmann et al., 2017; Sherrard et al., 2018). The RCM means that organisms can perceive MF signals through the quantum spin dynamics of a radical-pair reaction produced from the activation of cryptochrome (Cry), a kind of flavoprotein, also known as a blue light (BL) receptor in various organisms (Hore and Mouritsen, 2016), and this hypothesis has also been verified in *cry*-deficient *Drosophila melanogaster* who lose the response to MF (Gegear et al., 2008). However, the RCM fails to explain how *D. melanogaster* can sense the changes in the MF intensity and orientation (Hore and Mouritsen, 2016).

Recently Qin et al. (2016) claimed that they have found a homologous protein of the bacterial iron-sulfur cluster (ISC) assembly ISCA1, called a magnetic receptor (MagR), in *Homo sapiens*, *D. melanogaster*, and other species, which can combine with Cry to form a protein complex (MagR-Cry), namely a biological compass, to sense the changes in direction of MF, thus providing a new clue to explore the mechanism of biologically sensing MF. The ISC protein widely exists in *Homo sapiens*, animals, plants, and microorganisms (Wang H. X. et al., 2016) and possesses many biological functions, such as maintaining mitochondrial stability (Jensen and Culotta, 2000; Kaut et al., 2000; Pelzer et al., 2000), regulating iron ion binding and iron homeostasis in *Saccharomyces cerevisiae* (Krebs et al., 2001; Ding et al., 2005), and adjusting the circadian rhythm of animals (Kosmidis et al., 2011; Mandilaras and Missirlis, 2012). However, up to now, the role of ISC magneto-sensing in filamentous fungi has not been reported.

*Monascus* spp., which are edible filamentous fungi and can produce abundant secondary metabolites (SMs), such as *Monascus* pigments, monacolin K, citrinin, and so on (Chen et al., 2017), have been used for nearly 2,000 years in the world, especially in China, Japan, and other Asian countries (Chen et al., 2015). Previous studies have revealed that almost all fungi can sense and receive light signals through light receptors, such as green-light receptors, red-light receptors, and BL receptors (Schumacher, 2017). Among them, the BL receptor, Cry is the best-studied light receptor in fungi until now (Casas-Flores and Herrera-Estrella, 2016).

Recently, our and other research groups have discovered that BL and MF have significant effects on SMs production of *Monascus* spp. (Zhang et al., 2015; Wang L. et al., 2016; Wan et al., 2017). So *Monascus* spp. may exist a protein complex, such as MagR-Cry, to respond to the magnetic and light signals, and we have also discovered a *magR* gene in *M. ruber* M 7, hereinafter referred to *mr-magR*, but we did not find any homologous gene similar to *cry* or other BL receptor genes in the M7 genome that commonly appear in other fungi. Thus, we put forward a hypothesis that there might be an unknown BL receptor in *M. ruber* M7, namely Mr-BLR, to form a Mr-MagR-BLR complex to sense MF and BL signals. In order to explore this hypothesis, we firstly investigated the effects of BL, MF, and a combination of MF and BL (MF-BL) on the growth and morphological characteristics of M7, respectively, and found that MF-BL had the most significant effects on the M7 strain. Then, we searched the genomes of animals, plants, and microorganisms based on MagR of *D. melanogaster* (dMagR), and summarized a total of 73 proteins’ amino acid (AA) sequences with a dMagR similarity greater than 55% to construct a phylogenetic tree and analyzed AA sequences of Mr-MagR. After that, the full-length cDNA sequence of *mr-magR* was cloned, analyzed, and expressed in *Escherichia coli*, and Mr-MagR protein was purified by a Ni^+^-NTA column and identified by Western blot.

The abovementioned results have laid a foundation to seek Mr-BLR and investigate the relationship between Mr-MagR and Mr-BLR, which might exist in M7. According to a literature search, it is the first time to report the *magR* gene in filamentous fungi.

## Materials and Methods

### Strains and Plasmids

*M. ruber* M7 (CCAM 070120, Culture Collection of State Key Laboratory of Agricultural Microbiology, Wuhan, China) is stored in our laboratory (Chen and Hu, 2005). The pET-28a plasmid is deposited in our laboratory, too. *E. coli* [DH5α and BL21(DE3)] competent cells were purchased from TransGen Biotech Co., Ltd. (Beijing, China) and cultured in Luria-Bertani (LB) medium supplemented with 50 μg/mL kanamycin or ampicillin when required.

### Effects of MF, BL, and MF-BL on Morphologies of *M. ruber* M7

**FIGURE 1:**
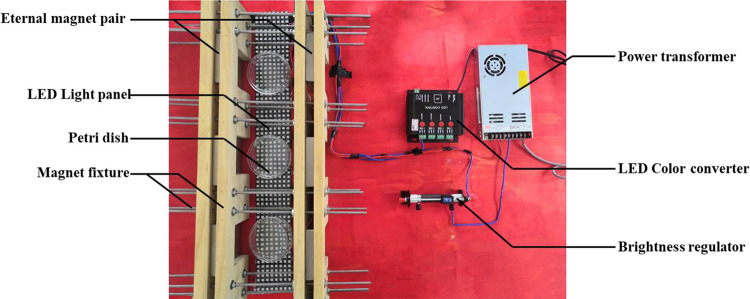
The photo of the device coupling lights and magnetic fields.

In order to investigate the effects of MF, BL, and MF-BL on M7, we have built a device of coupling light and MF (Figure 1). In the device, two permanent magnets are clamped by fixtures to form a magnet pair, and the magnetic flux densities between the two magnets can be controlled by adjusting the distance of the magnets; meanwhile, a light-emitting diode (LED) panel is placed on the bottom of the device, and the LED’s BL (465–467 nm) and its intensities can be monitored by the control system, including a power transformer, an LED color converter, and a brightness regulator of the sliding rheostat (Figure 1). The magnetic densities are detected with a Gauss meter (SJ700, Senjie Technology Co., Ltd., Guilin, China), and the BL intensities are measured by a light meter (VICTOR 1010A/D, Shengli Gao Electronic Technology Co., Ltd., Shenzhen, China). We confirmed that the intensity of MF in this study did not change the intensity of BL emitted by the LED.

After the M7 strain is cultured on potato dextrose agar (PDA) for 15 days at 28°C, its spores are harvested and adjusted to 1.0 × 10^6^ spores/mL with sterile water. Then 100 μL spore suspension is spread and inoculated onto PDA. Finally, PDA plates are put in the middle between the two permanent magnets (Figure 1) and incubated at 28°C for 10 days alone under 5, 10, and 30 mT MF by turning off BL, 500 lux BL by removing MF, and MF-BL by turning on the BL, respectively, to investigate the effects of MF, BL, and MF-BL on the colonial and microscopic morphologies of *M. ruber* M7 compared with the control (CK, neither BL nor MF).

Three parallel experimental groups are conduct for each condition. Colony diameter are defined as: Take two straight lines on the colony pass through the center of the colony, take the average value of the two straight lines. Calculate the standard deviation of the colony diameter of three parallel experiments.

### Bioinformatics Analysis of Mr-MagR in *M. ruber* M7

#### Phylogenetic Analysis of Mr-MagR

Based on AA sequences of the MagR (*D. melanogaster*), we used NCBI-BLASTP to search the genomes of *Homo sapiens*; animals; plants; filamentous fungi, including *Monascus* spp., *Saccharomyces cerevisiae*, *Actinomyces* spp. and bacteria to find the MagR protein. MagRs, which are homologically similar with dMagR higher than 55%, were selected to draw a phylogenetic tree by MEGA 7 (Kumar et al., 2016) and iTOL 4.0 with the maximum likelihood method (Letunic and Bork, 2019). Then several bioinformatics tools, including NCBI-CDD (Marchler-Bauer et al., 2016), Pfam (El-Gebali et al., 2018), and DNAMAN software, were applied to analyze the conserved domains and conserved AA sites of MagR proteins.

#### The Structure and Property Analyses of Mr-MagR

Softberry^1^ and NCBI ORF-Finder were used to compare and analyze the numbers of introns and the open reading frame (ORF) of the *mr*-*magR* gene. The hydrophilicity, physical and chemical properties, transmembrane region, signal peptide, subcellular localization, and secondary structure analysis of Mr-MagR were predicted and analyzed by the ExPASy-Prot Param tool and ExPASy-ProtScale (Gasteiger et al., 2005), TMHMM Server v. 2.0^2^ and TMpred Server^3^, SignalP 3.0 Server (Bendtsen et al., 2004), Psort II Prediction (Horton and Nakai, 1997), and PredictProtein and Sopma, respectively.

### Mr-MagR Heterologous Expression in *E. coli*

#### Construction of the Expression Vector for Mr-MagR

After RNA was extracted from M7 mycelia grown on PDA for 96 h at 28°C according to the manufacturer’s protocol (EZ-10 Spin Column RNA Purification Kit, BBI Markham, Canada), the full-length cDNA of *mr-magR* was achieved according to the rapid amplification of cDNA end (RACE) method previously reported (Yang et al., 2012). The full-length cDNA of *mr-magR* was sequenced (Sangon Biotech Co., Ltd., Shanghai, China), and its largest ORF was analyzed with ORF-Finder. After that, we designed the primers and the restriction sites with protective bases. Then the cDNA of M7 was used as a template to amplify *mr-magR*, and the final products were double-digested with corresponding restriction endonucleases and ligated to the pET-28a plasmid treated with the same digestion to get the expression vector. Finally, the expression vector (pET-28a-*mr-magR*) was transformed into *E. coli* DH5α competent cells by a chemical transformation method for enrichment and preservation. The nested primers used in the RACE method and the primers used to construct the expression vector are shown in Table 1.

**TABLE 1:** Primers used for RACE method and construction of expression vectors.

**Primer names**	**Sequences (5′–3′)**	**Functions**
3′connector 1	GCTGTCAACGATACGCTACGTAACGGCATGACAGTGTTTTTTTTTTTTTTTTTT	3′RACE first round of PCR amplification
3′connector 2	GATACGCTACGTAACGGCATGACAG	3′second round of PCR amplification
5F02	GACTCGAGTCGACATCGACCCCCCCCCCCCCCCCC	5′RACE PCR amplification
5′Mr-MagR GSP01	GCCTCTCCCTCTCTCACGCTAA	5′nested PCR amplification
5′Mr-MagR GSP02	GGTAAGGTGTGAGGACGATAAG	5′ nested PCR amplification
3′Mr-MagR GSP1	TGGGTGTGAAGAATCGGGGCTG	3′nested PCR amplification
3′Mr-MagR GSP2	ATGGGTTTTACTTGGGGCGTCT	3′nested PCR amplification
Mr-MagR-His-up-*Bam*HI	CGCGGATCCATGTCGTTTTGCTGCACCGTT	*mr-magR* 5′amplification
Mr-MagR-His-do-*Xho*I	CCGCTCGAGAACCATGAAAGATTCACCGCATC	*mr- magR* 3′amplification

**The underlined part are double-cleavage sites, GSP01 can amplify 323 bp of the 5′ end of the known cDNA sequence, GSP02 can amplify 178 bp of the 5*′ *end of the known cDNA sequence; GSP1 can amplify 332 bp 3*′ *end of the known cDNA sequence, GSP2 can amplify 97 bp of the 3*′ *end of the known cDNA sequence.**

#### Mr-MagR Expressed in *E. coli*

The pET-28a-*mr-magR* plasmids were transformed into *E. coli* BL21 (DE3), and the expression strains were verified by colony PCR. The expression strains (pET-28a-*mr- magR*-BL21) were incubated in LB medium containing kanamycin. When *OD*_600__*nm*_ value of the bacterial cells was up to about 0.6, they were treated with 0 (CK), 0.1, 0.2, 0.5, 1, 2 mM isopropyl β-D-1-thiogalactopyranoside (IPTG) overnight at 15°C to explore the optimal inducer concentration. The cells were harvested by centrifugation at 12,000 × g, 4°C, then resuspended in the lysis buffer (10 mM phosphate buffer saline (PBS), pH 7.5, 15 mM dithiothreitol) containing a complete protease inhibitor cocktail (CWBIO Biotechnology Co., Ltd., Beijing, China) and lysed by sonication on ice. The supernatant and precipitation of the lysate were collected through centrifugation, and ran SDS-PAGE, respectively.

#### Renaturation, Purification, and Western Blot Identification of Mr-MagR

Because Mr-MagR was expressed in *E. coli* BL21 (DE3) as the inclusion body (see results), after the bacterial cells were harvested and resuspended in the lysis buffer on ice, the inclusion body was washed twice with the PBS, 1% TritonX-100, 5 mM EDTA (pH 7.5), resuspended in PBS (pH 7.5) and washed to remove EDTA by centrifugation. The samples were diluted to the appropriate concentration (10 mL lysis buffer per gram of wet cells) in 6 M urea PBS to dissolve the precipitate, and we added 15 mM dithiothreitol to open the disulfide bond for 1 h at 25°C. Then, PBS with 0.3 mM oxidized glutathione and 3 mM reduced glutathione redox system (GSH-GSSG system) was configured as dialysate to promote the correct formation of disulfide bonds and to initiate the oxidative refolding. It should be noted that the appropriate volume of dialysate needs to be calculated to make the urea concentration equal to 4 M before the first time changing the dialysate. Then, the dialysate should be renewed every 8 h, and the final urea concentration should be 2 M and 1 M in order by calculating the volume added. During this process, the target protein Mr-MagR was allowed slowly into a thermodynamically stable structure at 4°C and we got the soluble Mr-MagR protein.

The soluble Mr-MagR was purified with Ni^2+^-NTA (Sangon Biotech Co., Ltd., Shanghai, China), and ran SDS-PAGE. Then it was transferred to PVDF membrane to conduct Western blot identification (Shen et al., 2018).

## Results

### Effects of MF, BL, and MF-BL on Morphologies of *M. ruber* M7

In order to investigate the effects of MF, BL, and MF-BL on the growth of M7, we used different intensities of MF (5, 10, and 30 mT), 500 lux BL, and their combination (MF-BL) to, treat the M7 strain in the magnetic-light device, respectively (Figure 1).

**FIGURE 2:**
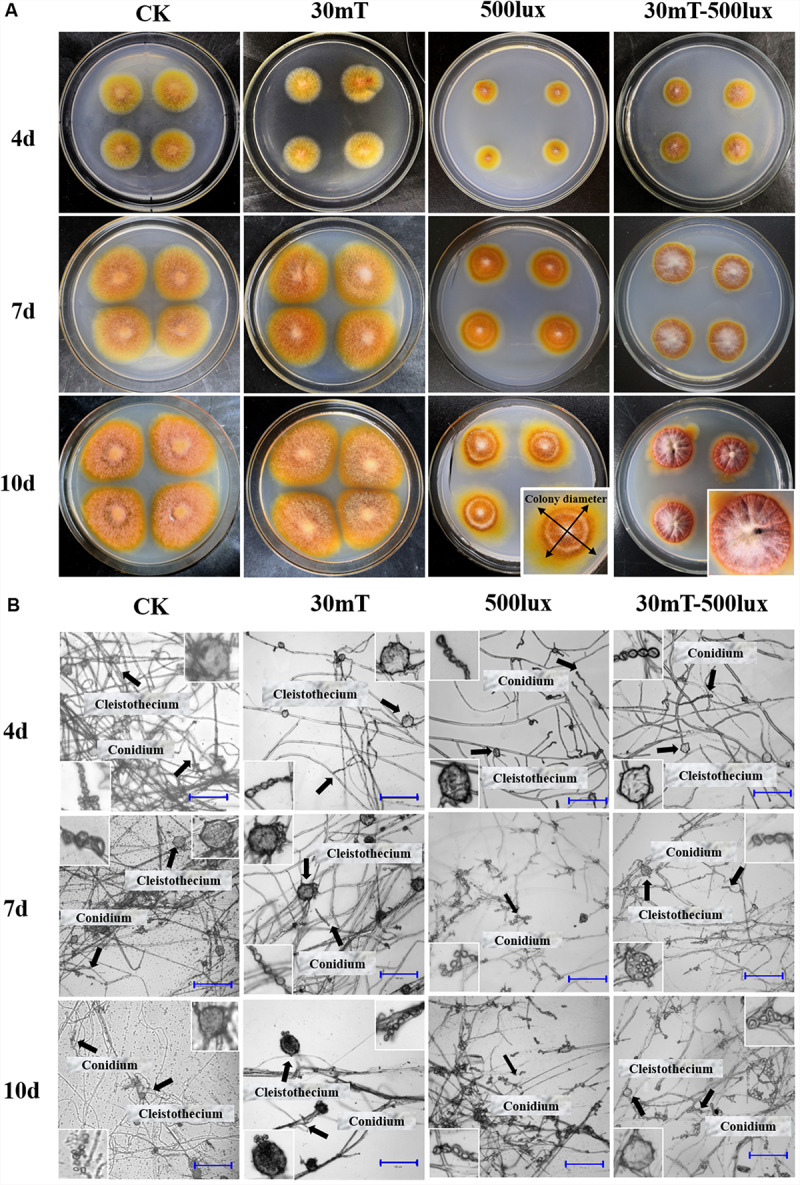
Growth of *M. ruber* M7 under CK, MF, BL, and MF-BL conditions. **(A)** Colony morphologies of *M. ruber* M7 under CK, 30 mT MF, 500 lux BL, and 30 mT-500 lux MF-BL conditions; **(B)** micromorphology of *M. ruber* M7 under CK, 30 mT MF, 500 lux BL, and 30 mT-500 lux MF-BL conditions. 5 mT and 10 mT conditions were almost same as 30 mT, so only 30 mT pictures were shown here.

The results of the 5 and 10 mT conditions were almost same as 30 mT, so only 30 mT pictures are shown here. The details of colonial and microscopic morphologies are described in Table 2 and Figure 2.

**TABLE 2:** Effects of 30 mT MF, 500 lux BL, and 30 mT-500 lux MF-BL on morphologies on *M. ruber* M7 (10 days).

**Treatment**	**Colonial characteristics**			**Asexual generation**	**Sexual generation**
	M (Φ*)**± σ**	**Mycelia**	**Colonial description**		
CK**	30 ± 0.47	Velvet-like, white aerial hypha.	The colony bulged with radiating patterns and pink-red overall. Light pink spores and aerial mycelia were distributed throughout the colony.	Conidia in chains.	Cleistothecia can be formed, and they burst to release ascospores.
MF	33 ± 2.05	Same as CK.	Same as CK.	Same as CK.	Same as CK.
BL	22 ± 1.63*	Flocculent interlaced, trace white aerial mycelia.	The colonies were slightly bulging, orange overall, and have three concentric circles: a large number of white aerial mycelia and spores were accumulated in the center area, and the first circle near the center was orange-yellow, light in color, and almost no mycelia; the second circle was formed almost entirely of white mycelia and spores and was macroscopically white; the outermost circle was darker orange-red with jagged edges.	A large number of conidia in chains or freely.	Cleistothecia cannot be formed after 7 days.
MF-BL	22 ± 0.81*	Many flocculent and velvety white mycelia were staggered.	The colonies were volcanic-like highly bulging, with a waxy edge and rough surface with darker red, and a large number of white aerial hyphae adhering to the surface.	Same as CK.	Same as CK.

Φ*: Colony diameter/mm of 10 days; values from the average of three parallel groups. **σ**: Standard deviation, values from the standard deviation of the colony diameters of the three parallel groups. *Significantly different (P < 0.05). CK**: Control check, in 28°C, dark condition, neither BL nor MF.

The results (Table 2 and Figure 2) showed that, compared with CK, the different densities of MF tested in this study have no effect on the M7 growth, but 500 lux BL significantly inhibited the colonial growth of M7, under which the colonies looked like concentric circles with less and white aerial hyphae (Figure 2A). It is worth mentioning that, at 500 lux BL, small and immature-like cleistothecia could be clearly observed only when M7 was cultured for 4 days; subsequently, the numbers of cleistothecia decreased significantly, and at 7 days, the cleistothecia could not be observed anymore (Figure 2B).

Under MF-BL, however, the colonies were obviously bulging, volcanic-like with folds and a lot of white aerial hyphae, and the small and immature-like cleistothecia were observed throughout the culture period from 4 to 10 days, indicating that the MF-BL can restore the partial growth of M7 and its sexual generation, which were inhibited by 500 lux BL (Table 2 and Figure 2).

### Bioinformatics Investigation of Mr-MagR in *M. ruber* M7

**FIGURE 3:**
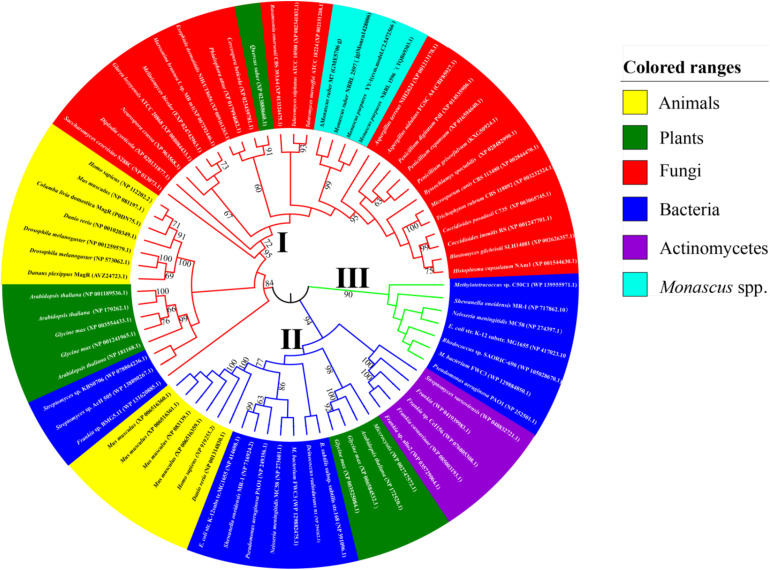
Phylogenetic trees of MagR. The AA sequence of the MagR of *D. melanogaster* published in NCBI as a control were aligned with the genome of four strains of *Monascus* spp. (*M. ruber* M7, *M. ruber* NRRL 2597, *M. purpures* YY-1, *M. purpures* NRRL 1596) and model organisms in animals, plants, and microorganisms in the Protein-NCBI database. A total of 73 MagRs with a homology ≥ 55% were selected and constructed with MEGA 7.0. The value next to the branch indicates the bootstrap support rate of the branch, and the bootstrap value was 1000 (Felsenstein, 1985). The evolution of Mr-MagR was estimated by the maximum likelihood method (Felsenstein, 1981). *M. ruber* M7 was marked by Δ.

Based on the AA sequences of dMagR, we searched the genomes of animals, plants, and microorganisms, including *Monascus* spp., from NCBI databases using BLASTP and summarized a total of 73 proteins’ AA sequences with a dMagR similarity greater than 55% and constructed a phylogenetic tree and analyzed AA sequences (Figure 3).

#### Phylogenetic Analysis of Mr-MagR

We find that MagR is ubiquitous in different species, and some organisms possess more than one MagR, indicating that the biological function of MagR may be highly conserved and indispensable in a wide range of organisms (Figure 3).

The MagR phylogenetic tree can be divided into three evolutionary branches as a whole when the bootstrap value is 1000. In Branch I, all MagRs are from bacteria. In Branch II, all MagRs are from animals, plants, and bacteria. In Branch III, MagRs come from animals, plants, fungi, and some bacteria, including MagRs from the *Monascus* species, which were clustered into a subcluster with 90% similarity and the closest to the MagR from *Aspergillus terreus* with 75% similarity (Figure 3). It is very interesting that MagRs in the same species may belong to different branches, indicating that different MagRs in the same species may have different ancestry. For example, *Glycine max* possesses four homologous sequences of MagR: two of them are in Branch II, and the others are in Branch III.

A gene, GME5706 g in the *M. ruber* M7 genome, for which the E value is 7E^–37^, 972 bp, containing two introns and three exons, with the highest match with *dmagR* was found, hereinafter named *mr-magR* (*magR* of *M. ruber* M7). The gene most similar to *mr-magr* is TQB69303.1, which was identified as an ISC assembly protein in the *M. purpureus* 1596 genome. The conserved domain analysis has revealed that Mr-MagR is an ISC assembly accessory protein belonging to the IscA/HesB superfamily (Zheng et al., 1998), and the AA sequence 142–244 is a highly conserved domain of IscA/HesB.

**FIGURE 4:**
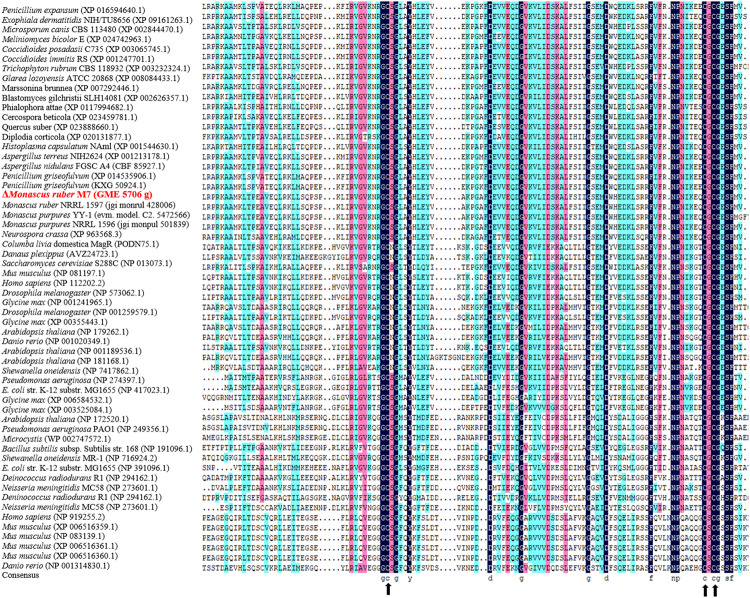
Conservative analysis of AA sequence of Mr-MagR homologous gene. The AA homology is identified by different colors: black for 100% homology, pink for ≥ 75%, and bright blue for ≥ 50%. Black arrows indicate highly conserved cysteine (Cys) residue positions.

In Mr-MagR, especially 3 cysteine (Cys, 172, 236, 238) and 5 Glycine (Gly, 171, 174, 196, 211, 239), 1 tyrosine (Tyr, 177), 1 aspartic (Asp, 189, 214), 2 phenylalanine (Phe, 224, 242), 1 asparagine (Asn, 229), 1 proline (Pro, 230), and 1 serine (Ser, 241), are highly conserved with ones from other species (Figure 4). The highly conservative sites of Cys are mainly responsible for the assembly of iron–sulfur clusters (Mandilaras and Missirlis, 2012; Wang H. X. et al., 2016).

#### Structure and Property Analysis of Mr-MagR

We used different bioinformatics tools to further analyze the structure and property of Mr-MagR. The results (Table 3) show that Mr-MagR is an unstable basic protein with 27.10 kDa molecular weight and 10.05 of isoelectric point. And it is also a hydrophilic protein without a transmembrane region and signal peptide.

**TABLE 3:** Bioinformatics analysis of the AA sequence of Mr-MagR.

**AA No.**	MM*	IP**	**Arg + Lys/Asp + Glu**	IS index***	**GRAVYT**	EPM****
244	27106.00	10.05	38/21	59.99	−0.57	78.3%

MM*: Protein molecular mass. IP**: Isoelectric point. IS index***: Protein instability index. EPM****: Probability of protein expression in mitochondria.

**FIGURE 5:**
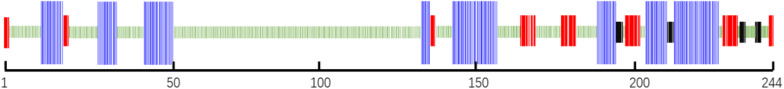
Secondary structure analysis of Mr-MagR. The blue parts represent 8 α-helix structures, the red parts represent 8 β-sheet structures, the black parts represent 4 β-turn structures, and the remaining green parts are random coil structures.

The secondary structure prediction of Mr-MagR shows that there are 24.59% of α-helix structure, 13.11% of β-sheet, and 3.69% β-turn structure, 58.61% random coil structure, including 8 α-helix, 8 β-sheet, 4 β-turn (Figure 5).

### Full-Length Clone and Heterologous Expression of *mr-magR* cDNA

#### Gene Clone and the Construction of Heterogeneous Expression Host

**FIGURE 6:**
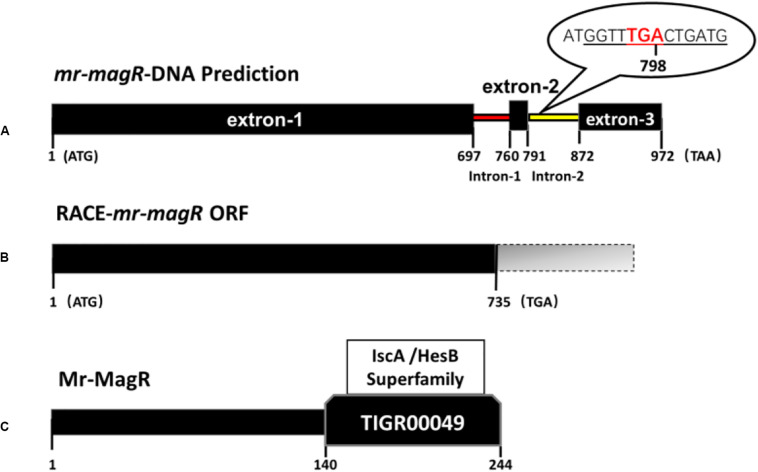
DNA, cDNA, and AA sequence information for *mr-magR.* **(A)** The prediction of the *mr-magR* gene is 972 bp, including two introns; the underlined part is the 5′-end partial sequence of intron 2; TGA is the stop codon. **(B)** *mr-magR* obtained by RACE-PCR amplification, and the ORF is 735 bp; **(C)** NCBI-CDD analysis shows that Mr-MagR belongs to the iron–sulfur cluster assembly accessory protein of the IscA/HesB superfamily, and the 142–244 AA are conserved domains.

We obtained untranslated DNA fragments: 127 bp at the 3′-end and 187 bp at the 5′-end of *mr-magR* cDNA by two rounds of RACE-PCR based on the primers in Table 1. After adding the predicted 972 bp, we got the full-length cDNA of *mr-magR* with 1,286 bp. Then, we analyzed this 1286 bp by ORF finder, and the results show that the actual ORF of *mr-magR* is only 735 bp (Figure 6B), which includes exon 1 (697 bp), exon 2 (31 bp), and the 3′ end (7 bp) of intron 2, indicating that intron 2 is not a true intron. The exon 3 was not included in the actual ORF due to the presence of the stop codon TGA at the 3′ end of intron 2 (Figure 6A). Therefore, the maximum ORF of *mr-magR* is 735 bp, encoding 244 AA.

After the *mr-magR* gene with 735 bp was cloned, its expression vector with His-tag, fused to the N-terminal was constructed and expressed in *E. coli* BL21 (DE3).

#### Expression, Renaturation, Purification, and Identification of Mr-MagR Protein

**FIGURE 7:**
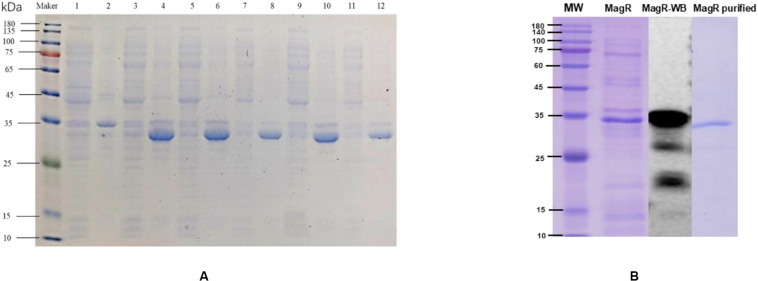
Induced expression, purification, and WB verification of Mr-MagR protein. **(A)** 1: Supernatant without inducer; 2: Precipitate without inducer; 3, 4: Supernatant and precipitate of 0.1 mM IPTG; 5, 6: Supernatant and precipitate of 0.2 mM IPTG; 7, 8: Supernatant and precipitate of 0.5 mM IPTG; 9, 10: Supernatant and precipitate of 1 mM IPTG; 11, 12: Supernatant and precipitate of 2 mM IPTG. **(B)** Purification and WB verification results of Mr-MagR protein.

After pET-28a-*mr-magR*-BL21 were incubated in LB medium containing kanamycin when the *OD*_600__*nm*_ value of the bacterial cells was up to about 0.6, the *E. coli* cells were induced with the 0.1 mM IPTG, 15°C to produce Mr-MagR, which was mainly expressed in the inclusion body by running SDS-PAGE (Figure 7A). So the denaturing conditions were optimized, and 6 M urea-PBS was selected to dissolve Mr-MagR in the inclusion bodies. Finally, we got the target protein, after Mr-MagR was dialyzed in an improved GSH-GSSG system with PBS at 4°C for 24–36 h, purified with a Ni-NTA column (Figure 7B), detected by SDS-PAGE and identified by Western blot.

## Discussion

The behavior of many animals, such as migratory birds, monarch butterflies, salmon, and lobsters to orientate and migrate over long distances was discovered in the last century (Matthews, 1951; Quinn, 1980; Boles and Lohmann, 2003; Cain et al., 2005; Zhan et al., 2011). To elucidate this phenomenon, many hypotheses have been proposed, among which the magnetite-based model and RCM (the radical-pair-reaction-based chemical model) are in-depth studies (Frankel and Blakemore, 1980; Ritz et al., 2000; Hore and Mouritsen, 2016; Nordmann et al., 2017). The main issue that currently exists in the magnetite-based model is that no magnetic particle has been found up to now. About RCM, cryptochrome (Cry) is thought to be a magnetic receptor (Hore and Mouritsen, 2016), and in 2016, a MagR-Cry protein complex was discovered as a biocompass, which can perceive orientation and intensity of magnetic fields (MF) (Qin et al., 2016). Although such biocompass still has controversies, it may provide a new clue in this field. Especially, the MagR-Cry complex couples the effects of MF and light on organisms.

In this study, we found that the MF-BL has a significant effect on the growth of *M. ruber* M7 (Figure 2), suggesting that M7 may be able to perceive magnetic and light signals. We also searched the *mr-magR* gene but did not find any gene homologous to the BL receptor including *cry* in the M7 genome (Wang L. et al., 2016). In order to explore if there exists any unknown BL receptor (Mr-BLR) in the *M. ruber* M7 genome and investigate interaction of Mr-MagR with Mr-BLR, we plan to use an improved His pull-down method to find the Mr-BLR from the whole M7 protein pool, which requires obtaining the Mr-MagR protein and using it to get the Mr-BLR protein through the protein–protein interaction method. So the results in this study will lay the foundation for finding a new BL receptor.

In current study, we have found that MF-BL can partially restore the growth and the sexual generation of M7, which are inhibited by 500 lux of BL although MF alone has no effect seemingly on M7 (Figure 2 and Table 2). We are doing more experiments to explain this result.

Bioinformatics analysis of *mr-magR* shows that it contains two introns (Figure 6A), but the full-length cDNA of *mr-magR* obtained by RACE-PCR showed that the gene only contains intron 1 (Figure 6B). Intron 2 may be involved in the expression of this gene through alternative splicing (Grützmann et al., 2013), which was common in eukaryotes. Therefore, we also designed primers and cloned and expressed the predicted *mr-magR* cDNA sequence in *E. coli* as well. The band size was the same as the protein expressed in this study. This result indicates that the 3′-end of intron 2 was indeed involved in gene transcription, confirming that it was indeed an exon. In order to initially explore the conditions for alternative splicing of this gene, we extracted total RNA from M7 at different culture times (3, 4, and 5 days) for RACE-PCR amplification, and the results showed that the gene does indeed only contain intron 1. These results indicated that RACE-PCR was necessary for the heterologous expression of eukaryotic genes.

To get soluble target protein, we truncated expression of the highly conserved domain of *mr-magR* (*mr-cdmagR*, AA NO.:142-244, see Figure 6C), to obtain the protein containing the core function of Mr-MagR. Then, we connected *mr-magR* and *mr-cdmagR* to the pGEX-GST-TEV and pATX-SUMO plasmid, respectively, which are commonly used dissolution tags, to induce expression. Yet the protein still existed as an inclusion body. We also performed a heterologous expression of Mr-MagR in *Pichai pastoris*. Neither Mr-MagR nor Mr-CDMagR were expressed in *P. pastoris*, suggesting that directly obtaining soluble Mr-MagR may require an expression system closer to the *M. ruber* M7, such as *Aspergillus oryzae* or *Aspergillus niger*.

## Data Availability Statement

The raw data supporting the conclusions of this article will be made available by the authors, without undue reservation, to any qualified researcher.

## Author Contributions

FC supervised the entire work, planned the experiments, and revised the manuscript. HZ performed the experiments, analyzed the data, and wrote the manuscript. SY guided HZ to conduct the experiments in molecular biology parts. All authors contributed to the manuscript and approved the submitted version.

## Conflict of Interest

The authors declare that the research was conducted in the absence of any commercial or financial relationships that could be construed as a potential conflict of interest.

## Footnotes

1 www.softberry.com

2 http://www.cbs.dtu.dk/services/TMHMM/

3 https://embnet.vital-it.ch/software/TMPRED_form.html
